# Patient-Reported Outcome Measures After Liver Resection: A Prospective Single-Center Analysis of Patient-Reported Outcomes and Predictors of Recovery

**DOI:** 10.3390/cancers18172767

**Published:** 2026-08-26

**Authors:** Matteo Colangelo, Marcello Di Martino, Teresa Gatto, Paolo Baroffio, Matteo Donadon

**Affiliations:** 1 Department of Health Sciences, University of Piemonte Orientale, 28100 Novara, Italy; 2 Division of General Surgery, University Hospital Maggiore della Carità, 28100 Novara, Italy

**Keywords:** liver surgery, hepatectomy, patient-reported outcome measures, PROMs, quality of life, hepatocellular carcinoma, colorectal liver metastases, cholangiocarcinoma

## Abstract

Background: Traditional outcome assessment after liver surgery mainly relies on perioperative morbidity and mortality. However, these parameters fail to capture the multidimensional recovery experienced by patients after hepatic resection. This study aimed to evaluate changes in quality of life (QoL) between the preoperative and three-month postoperative assessments using Patient-Reported Outcome Measures (PROMs) and to identify factors associated with impaired postoperative recovery. Methods: A prospective observational study was conducted including 71 consecutive patients undergoing liver resection at a tertiary hepatobiliary referral center between May 2024 and May 2025. QoL was assessed preoperatively and three months after surgery using the EORTC QLQ-C30 and QLQ-LMC21 questionnaires. PROMs were correlated with demographic, clinical, operative, and postoperative variables. Univariable and multivariable analyses were performed to identify factors associated with worse postoperative QoL. Results: Significant improvements in postoperative QoL were observed at three months compared with baseline in both the QLQ-C30 and QLQ-LMC21 questionnaires (both *p* < 0.001). Psychological domains also significantly improved after surgery. Patients with ASA scores ≥ 3 (*p* = 0.044) and those requiring intraoperative blood transfusion (*p* = 0.011) reported significantly worse postoperative quality-of-life scores. On multivariable regression analysis, Grade B bile leak was associated with impaired postoperative QLQ-C30 outcomes (95% CI 0.75–23.81; *p* = 0.038). Conclusions: Liver resection was associated with improvement in patient-reported QoL three months after surgery. PROMs provide clinically relevant information beyond conventional surgical metrics and may help identify patients requiring tailored perioperative support. The integration of PROMs into liver surgery pathways may contribute to the development of a more patient-centered model of surgical care.

## Simple Summary

Traditionally, outcomes after liver surgery have been evaluated using clinical indicators such as mortality, complications, and length of hospital stay. However, these parameters do not fully capture how patients perceive their recovery and quality of life (QoL) after surgery. In this study, we prospectively evaluated patient-reported outcomes in 71 patients undergoing liver resection using validated quality-of-life questionnaires. We observed significant differences in both physical and psychological domains between the preoperative and three-month postoperative assessments. Poorer postoperative QoL was associated with higher preoperative ASA scores and intraoperative blood transfusion. In multivariable analysis, Grade B bile leak emerged as a predictor of impaired recovery. These findings support the integration of patient-reported outcome measures (PROMs) into routine perioperative care to identify vulnerable patients early and personalize postoperative support pathways.

## 1. Introduction

Liver resection remains a potentially curative treatment option for a wide spectrum of benign and malignant hepatic diseases. Historically, hepatic surgery was associated with substantial morbidity and mortality, mainly due to intraoperative hemorrhage and postoperative liver failure [1,2,3,4]. However, advances in surgical technique, anesthetic management, imaging, perioperative care, and patient selection have progressively improved postoperative outcomes [5,6].

Since the second half of the 20th century, however, the introduction of increasingly precise operative techniques, driven by a deeper understanding of hepatic anatomy, has significantly reduced mortality rates associated with major liver resections. Concurrently, this has encouraged the implementation of radical but conservative “parenchymal-sparing” techniques [7,8,9]. These advancements have translated into a sharp decrease in post-hepatectomy liver failure (PHLF) and associated morbidity, particularly in patients with impaired liver function. Precise volumetric study of the future liver remnant (FLR) and quantitative assessment of liver function [10] have led to more rigorous patient selection, resulting in significantly improved surgical outcomes [11,12,13].

The widespread use of intraoperative ultrasound (IOUS) [14] and strategies for inducing liver hypertrophy, such as portal vein embolization (PVE) [15], liver venous deprivation (LVD) [16,17], and associating liver partition and portal vein ligation for staged hepatectomy (ALPPS) [18,19,20] have revolutionized the surgical approach. These innovations have not only drastically reduced PHLF rates but have also extended oncological survival by enabling iterative resections for intrahepatic recurrence. The systematic introduction of bleeding control techniques via selective vascular occlusion [21], the evolution of parenchymal transection devices [22], the implementation of minimally invasive (laparoscopic and robotic) surgery, and accurate 3D preoperative planning [23,24] have expanded resectability criteria to include previously inoperable patients. Furthermore, advances in intraoperative anesthetic management and Enhanced Recovery After Surgery (ERAS) protocols have improved results [25]. Low central venous pressure within a goal-directed fluid therapy strategy has replaced traditional pre- and intraoperative fluid loading, reducing blood loss and transfusion requirements [26,27]. Consequently, perioperative mortality in high-volume referral centers has been drastically reduced to approximately 1–2% [28,29,30].

Traditionally, the quality of liver surgery has been evaluated using objective clinical parameters such as postoperative morbidity, mortality, blood loss, length of hospital stay, and readmission rates. Although mortality rates remain standard indicators of surgical quality, they fail to effectively evaluate individual or institutional performance. This limitation arises from their inability to account for critical patient population variables, such as age and comorbidities, which significantly impact short- and long-term outcomes [5]. In 2008, the World Health Organization (WHO) highlighted the extensive implications of postoperative complications, which extend beyond clinical outcomes to generate significant socioeconomic consequences [31]. Quality improvement has emerged as a major global priority aimed at reducing costs while maintaining healthcare efficiency and accessibility. Continuous performance improvement policies have been widely adopted to deliver high-quality services to surgical patients, based on the premise that superior quality stems from the systematic analysis and enhancement of work processes [32]. More recently, composite metrics such as Textbook Outcome, Benchmark Values, and Failure-to-Rescue have been introduced to provide a more comprehensive evaluation of surgical quality [5,33]. Despite their clinical utility, these indicators do not fully capture the patient’s perception of recovery, functional status, and psychological well-being after surgery. A patient without major complications may still experience prolonged fatigue, pain, emotional distress, or delayed return to normal activities. Consequently, increasing attention has been directed toward patient-centered outcomes including perceived quality of life (QoL) [33].

As highlighted by the Outcome4Surgery Consensus [34], there is an increasing need for outcome indicators reflecting the patient’s perspective. Patient-Reported Outcome Measures (PROMs) and Patient-Reported Experience Measures (PREMs) represent valuable tools for evaluating health status directly from the patient’s perspective. While PREMs record perceptions of healthcare services [35], PROMs measure health status, symptoms, and functional recovery directly from the patient’s perspective without clinician interpretation [36]. Beyond their utility in benchmarking and research, PROMs hold substantial clinical potential when integrated into the care pathway in real time. Dynamic data collection can act as an early warning system; for instance, an unexpectedly high post-discharge score in the pain or fatigue domain can prompt early supportive intervention by the surgical team, thereby preventing readmissions or secondary complications that could otherwise be managed in an outpatient or home setting [37]. PROMs shift postoperative monitoring from a passive to a proactive process, allowing early identification of subclinical issues, personalized rehabilitation strategies, and comparison between surgical approaches beyond conventional complication rates.

Although PROMs are well established in colorectal surgery [38,39], evidence in liver surgery remains heterogeneous and methodologically inconsistent [40,41,42,43]. The aim of this study was to evaluate changes in quality of life between the preoperative and three-month postoperative assessments using validated PROM instruments and to identify demographic, clinical, and surgical factors associated with impaired postoperative recovery.

## 2. Materials and Methods

### 2.1. Study Design and Patient Population

This was a prospective single-center observational cohort study including consecutive patients undergoing curative-intent liver resection performed by the Hepatobiliary Surgery team at the Division of General Surgery, University Hospital Maggiore della Carità, Novara, Italy, between May 2024 and May 2025.

Patients diagnosed with hepatocellular carcinoma (HCC), intrahepatic cholangiocarcinoma (iCCA), perihilar cholangiocarcinoma (pCCA), colorectal liver metastases (CRLMs), neuroendocrine liver metastases, sarcoma metastases, and benign liver lesions were included.

Clinical and operative data were extracted from a prospectively maintained institutional database and included demographic variables, preoperative clinical characteristics, operative details, postoperative outcomes, and oncological data.

The study was conducted according to the Declaration of Helsinki.

### 2.2. PROM Assessment

QoL was assessed preoperatively during the pre-admission visit and postoperatively three months after surgery using the European Organization for Research and Treatment of Cancer Quality of Life Questionnaire Core-30 (EORTC QLQ-C30) version 3.0 and the European Organization for Research and Treatment of Cancer Quality of Life questionnaire liver module (QLQ-LMC21).

The EORTC QLQ-C30 is a cancer-specific QoL assessment tool comprising five functional scales (physical, role, social, emotional, and cognitive functioning) and symptom-specific items (fatigue, nausea/vomiting, pain, dyspnea, insomnia, appetite loss, constipation/diarrhea), including the financial impact of the disease. It consists of 30 items: 28 scored on a 4-point scale and 2 on a 7-point grading scale [44,45].

The QLQ-LMC21 is a 21-item module (4-point scale) originally developed for patients with colorectal liver metastases. It evaluates liver-specific symptoms including pain, fatigue, nutritional issues, anxiety, social concerns, and disease-related distress. Although originally developed for colorectal liver metastases, the QLQ-LMC21 was selected to provide a liver-specific symptom assessment across the heterogeneous hepatic surgery population included in the present study [42].

Questionnaire responses were analysed using unweighted aggregate scores. For the QLQ-C30, the aggregate score was calculated for each patient by summing the raw responses to all 30 questionnaire items (QLQ-C30 aggregate score = Q1 + Q2 + … + Q30). Similarly, the QLQ-LMC21 aggregate score was calculated as the sum of the raw responses to all 21 items (QLQ-LMC21 aggregate score = LMC1 + LMC2 + … + LMC21). These aggregate measures were used to provide a single composite measure for longitudinal and exploratory analyses and should not be interpreted as the validated EORTC QLQ-C30 or LMC21 Summary Score as previously reported in the literature [46,47,48,49].

Secondary exploratory analyses were performed using selected questionnaire items addressing psychological and psychosocial aspects as defined in the EORTC QLQ-C30 and QLQ-LMC21 domains. For the QLQ-C30, an exploratory psychological/psychosocial item aggregate was calculated as the sum of items 21–28. For the QLQ-LMC21, the aggregate was calculated as the sum of items 15–20.

### 2.3. Preoperative Assessment and Surgical Management

Diagnoses were established according to current international guidelines [42]. All patients underwent preoperative laboratory testing, including tumor markers (AFP, CEA, and CA19-9), and imaging assessment with multiphasic contrast-enhanced computed tomography (CT) and/or magnetic resonance imaging (MRI) with hepatospecific contrast agents; 18 fluorodeoxyglucose positron emission tomography (FDG-PET) and percutaneous biopsy were performed when clinically indicated.

Preoperative future liver remnant (FLR) was estimated via CT or MRI volumetry. For major hepatectomy, a minimum FLR of 25% was required in patients with normal liver function and 40% in patients with chronic liver disease or previous extensive chemotherapy. Portal and hepatic vein embolization were considered in patients with inadequate FLR for staged liver resections. Major hepatectomy was defined as resection of three or more liver segments. Surgical management was performed according to previously described principles, while perioperative blood management followed established criteria, as reported elsewhere [43].

### 2.4. Statistical Analysis

Data were analyzed using STATA^®^ version 26 (StataCorp LP, College Station, TX, USA). Continuous variables are presented as median and range according to distribution. Categorical variables are expressed as frequencies and percentages. Comparisons between QLQ-C30 and QLQ-LMC21 aggregate scores obtained preoperatively and 3 months after surgery were performed using the Wilcoxon signed-rank test for paired observations. Multivariable linear regression analysis was performed to identify independent predictors associated with postoperative QoL. Variables with *p* < 0.10 at univariable analysis, together with variables considered clinically relevant based on their potential association with postoperative recovery, were included in the multivariable model. A *p*-value < 0.05 (two-tailed) was considered statistically significant.

## 3. Results

### 3.1. Patient Characteristics

A total of 71 consecutive patients undergoing liver resection with curative intent were included. As shown in Table 1, median age was 72 years (range 39–87), and 41% of patients were female. Most patients had preserved functional status, with only 4% presenting an ECOG Performance Status ≥ 2. Median Charlson Comorbidity Index was 7, while 24% of patients had an ASA score ≥ 3.

Colorectal liver metastases represented the most frequent indication for surgery (39%), followed by hepatocellular carcinoma (29%) and cholangiocarcinoma (12%). Cirrhosis and hepatic steatosis were present in 30% and 25% of patients, respectively. The median number of lesions was 1 (range 1–20), with a median lesion diameter of 3.7 cm. At the time of surgery 17 patients in the cohort (24%) had received prior liver-directed treatments, such as previous hepatic resections or thermal ablation therapies, including radiofrequency ablation (RFA) and microwave ablation (MWA).

### 3.2. Surgical Outcomes

Major hepatectomy was performed in 27% of cases, while 73% underwent minor liver resection. Anatomical resections accounted for 27% of procedures. Most patients underwent open surgery, whereas 17% were treated using minimally invasive approaches. Median operative time was 288 min. Intermittent hilar clamping was used in 83% of patients, with a median clamping duration of 48 min. Intraoperative blood loss > 500 mL occurred in 18% of patients, while 24% required intraoperative blood transfusion. Overall postoperative morbidity was 23%, whereas major complications (Clavien–Dindo 3–4) occurred in 6% of patients. A total of 5 (7%) of patients presented bile leak grade B. No patient developed post-hepatectomy liver failure, and 90-day mortality was 0.

### 3.3. Changes in PROMs

**Figure 1:**
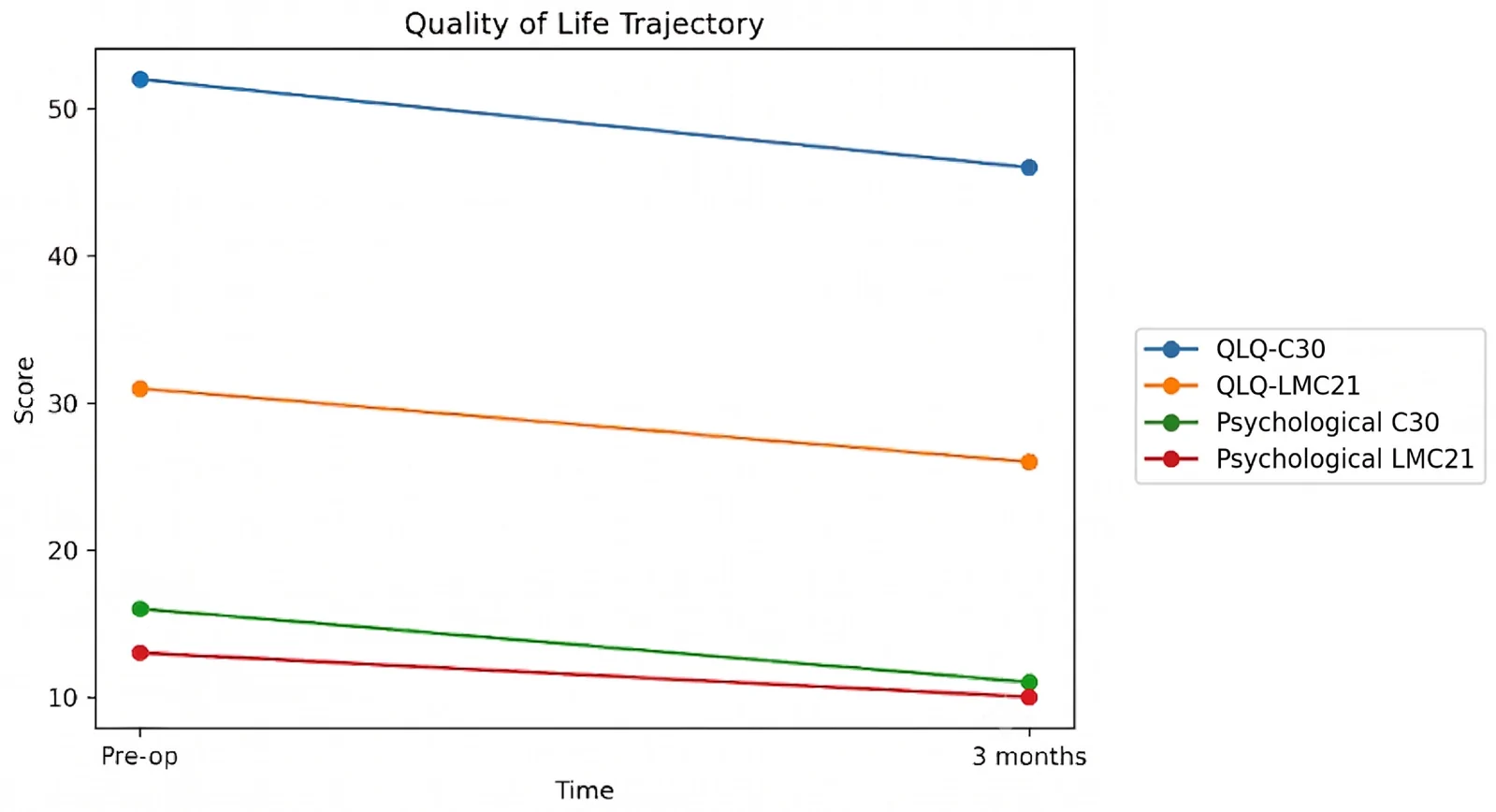
Changes in median EORTC QLQ-C30 and QLQ-LMC21 aggregate scores and psychological domain median aggregate scores between baseline and the three-month postoperative assessment. Significant reductions were observed at three months in both aggregate and psychological scores.

All 71 patients included in the study completed both the EORTC QLQ-C30 and QLQ-LMC21 questionnaires at baseline and at the three-month postoperative assessment. Therefore, the questionnaire completion rate was 100% at both time points, with no missing questionnaire data or loss to follow-up. Both QLQ-C30 and QLQ-LMC21 aggregate scores differed significantly between baseline and the three-month postoperative assessment. Median QLQ-C30 aggregate scores decreased from 52 preoperatively to 46 at three months (*p* < 0.001), while median QLQ-LMC21 aggregate scores decreased from 31 to 26 (*p* < 0.001) (Table 2). Similarly, differences between the two assessments were observed in the psychological domains of both questionnaires (both *p* < 0.001). Figure 1 illustrates the changes in quality-of-life scores between baseline and three months.

### 3.4. Predictors of Worse Postoperative QoL

Patients with ASA scores ≥ 3 reported significantly worse postoperative QLQ-C30 aggregate scores compared with ASA I–II patients (*p* = 0.044). Similarly, intraoperative blood transfusion was associated with worse postoperative QoL outcomes (*p* = 0.011) (Supplementary Tables S1 and S2). No statistically significant differences in postoperative QoL were observed according to surgical approach, cirrhosis, steatosis, extent of resection, postoperative ascites, or major postoperative complications.

**Figure 2:**
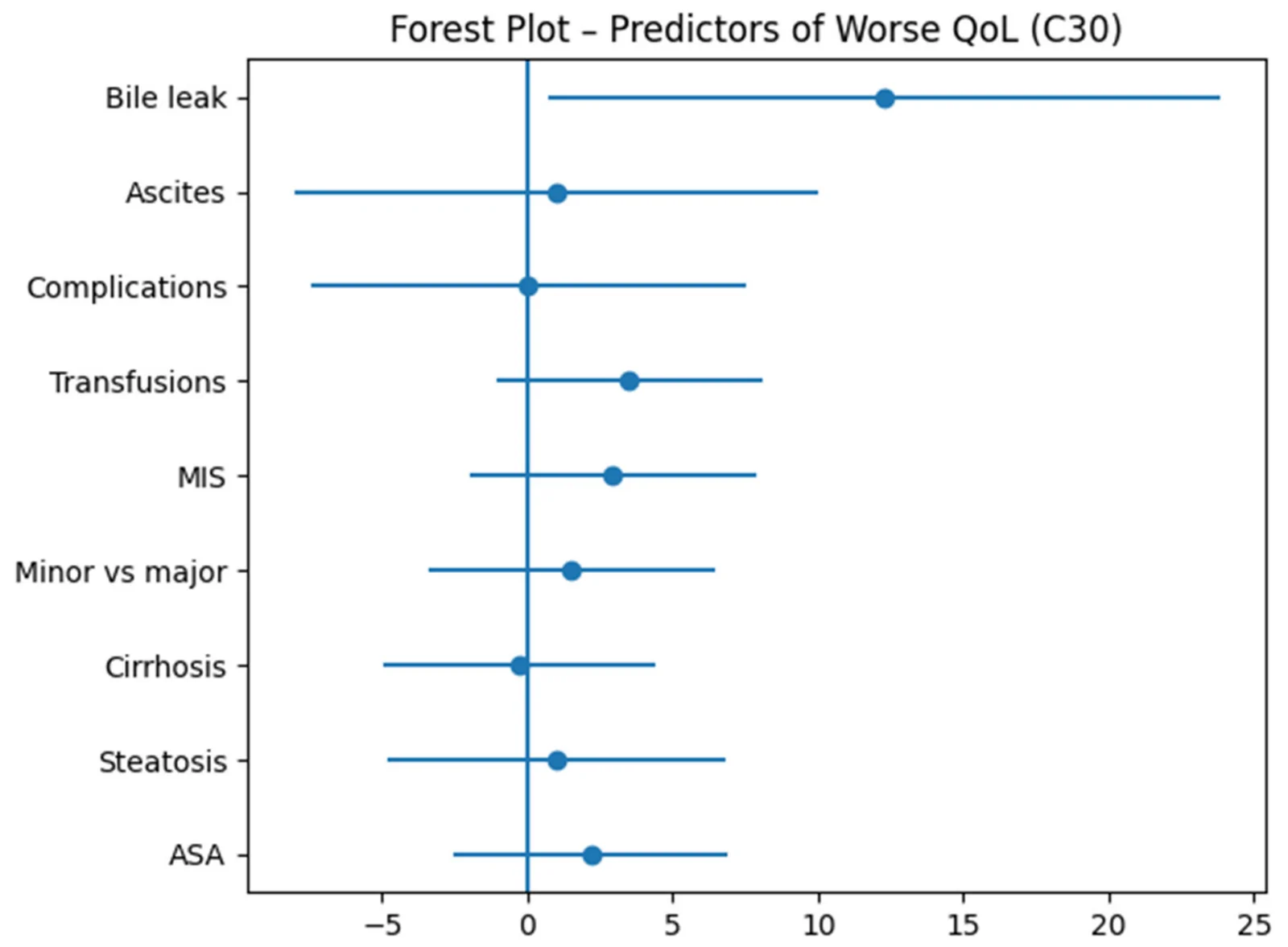
Forrest plot for Predictors of Worse Postoperative QLQ-C30 aggregate score on the adjusted analysis.

On multivariable linear regression analysis, Grade B bile leak was associated with worse postoperative QLQ-C30 outcomes (β = 12.28, 95% CI 0.75–23.81; *p* = 0.038). Table 3 and Table 4 and Figure 2 summarize the predictors associated with worse postoperative QoL.

### 3.5. Predictors of Worse Postoperative QoL: Subgroup Analysis Focused on Psychological Outcomes

Subgroup analysis focused on psychological domains demonstrated significantly worse postoperative QLQ-LMC21 psychological aggregate scores among patients with ASA scores ≥ 3 (*p* = 0.043). Major liver resection was associated with impaired postoperative psychological outcomes in both the QLQ-C30 (*p* = 0.008) and QLQ-LMC21 questionnaires (*p* = 0.035). Additionally, perioperative blood transfusion was associated with worse postoperative QLQ-C30 aggregate scores (*p* = 0.039) (Supplementary Tables S3 and S4).

## 4. Discussion

The present study demonstrated significant differences in patient-reported QoL between the preoperative and three-month postoperative assessments across both the EORTC QLQ-C30 and QLQ-LMC21 questionnaires. In addition to conventional oncological and technical outcomes of hepatic surgery, these findings highlight the importance of integrating patient-centered metrics into perioperative evaluation.

The better QoL scores observed at three months compared with baseline may reflect several factors, including recovery from the acute postoperative phase, treatment of the underlying disease, and changes in patients’ physical and psychological status over time. However, given the observational design and absence of a comparison group, the observed changes cannot be attributed specifically to liver resection. While previous studies have described a temporary decline in QoL during the early postoperative period following major surgery, the present analysis at three months likely reflects the consolidation phase of recovery [50,51,52]. At this stage, the negative impact of surgical stress on functional performance may have largely resolved, while the psychological benefits of disease treatment become more clinically evident.

The present findings suggest that preoperative functional reserve represents a key determinant of postoperative recovery. Specifically, patients with ASA scores ≥ 3 exhibited significantly impaired postoperative quality of life compared with ASA I–II patients. This observation is consistent with previous studies identifying frailty and comorbidity burden as predictors of slower or incomplete postoperative recovery [52,53,54].

The present analysis also identified intraoperative blood transfusion as a significant predictor of impaired postoperative quality of life. Intraoperative blood transfusion likely reflects increased operative complexity, greater blood loss, and higher surgical stress. These findings further support the importance of perioperative strategies aimed at minimizing surgical stress and blood loss, including enhanced anesthetic management, minimally invasive surgery, and parenchymal-sparing approaches [55,56,57,58,59].

Contrary to some previous reports, no statistically significant differences in 3-month quality of life were observed between patients with and without major postoperative complications. However, this finding should be interpreted cautiously given the relatively small number of severe complications in the present cohort. Nevertheless, multivariable linear regression showed an association between Grade B bile leak and impaired postoperative quality of life. This finding should be considered exploratory, given that only five patients experienced Grade B bile leak and the 95% CI was wide, indicating considerable uncertainty around the effect estimate. Although the association is clinically plausible, as bile leaks may require prolonged drainage, interventional procedures, antibiotic therapy, and delayed functional recovery, confirmation in larger cohorts is required.

The implementation of PROMs in hepatobiliary surgery remains heterogeneous. Existing studies frequently use different questionnaires, heterogeneous patient populations, and variable follow-up intervals, thereby limiting comparability across studies. The present study contributes additional prospective evidence supporting the feasibility and clinical utility of PROM integration into liver surgery pathways.

The integration of PROMs into postoperative management pathways may facilitate early supportive interventions and personalized postoperative care. Furthermore, preoperative psychological PROM domains may help identify vulnerable patients who could benefit from tailored psycho-oncological support and closer perioperative monitoring. These findings support the progressive integration of PROMs into perioperative liver surgery care, in line with the recommendations proposed by the recent Outcome4Surgery Consensus [34]. In particular, psychological PROM items may help identify patients experiencing elevated levels of distress who could benefit from early psycho-oncological intervention [60,61,62]. The effective implementation of PROM-based perioperative care pathways will likely require dynamic collection systems integrated within postoperative routine clinical workflows [25]. Lastly, this kind of information is very important to improve and make counseling with patients and their families more effective when it is necessary to anticipate a complex surgical pathway that may be associated with risks. Having similar studies collecting PROM data directly from patients represents valuable evidence that can make a difference in supporting one treatment pathway rather than another.

The present study has several limitations. First, this was a single-center study with a relatively limited sample size. Second, the cohort included different liver diseases and surgical indications representing a source of clinical heterogeneity, as preoperative psychological status, previous treatments, prognostic awareness, and disease-related expectations may differ substantially across diagnostic groups and potentially influence patient-reported QoL. Given the sample size and the relatively limited number of patients within individual disease categories, disease-specific analyses would not provide sufficiently robust estimates and could lead to unreliable conclusions. Third, for the limited number of patients and of events we elected not to apply penalized regression of internal validation procedures, as we believe that the limited sample size would not allow a sufficiently robust assessment of model performance or generalizability. Fourth, postoperative assessment was limited to a three-month follow-up period, preventing evaluation of long-term trajectories. Fifth, the QLQ-LMC21 questionnaire was originally developed for colorectal liver metastases and may not fully capture disease-specific symptoms across all hepatic pathologies. Finally, the overall QLQ-C30 and QLQ-LMC21 outcomes were analysed using exploratory unweighted raw-item aggregate scores rather than the formally validated EORTC QLQ-C30 Summary Score. Although aggregate approaches have precedent in the literature, the measures used in the present study are not independently validated composite scores and their results should therefore be interpreted as exploratory.

Despite these limitations, the study provides clinically relevant prospective data supporting the integration of PROMs into perioperative liver surgery care. Future multicenter studies should aim to define standardized PROM protocols, clinically meaningful thresholds with specific time frames, and PROM-driven intervention algorithms.

## 5. Conclusions

**Figure 3:**
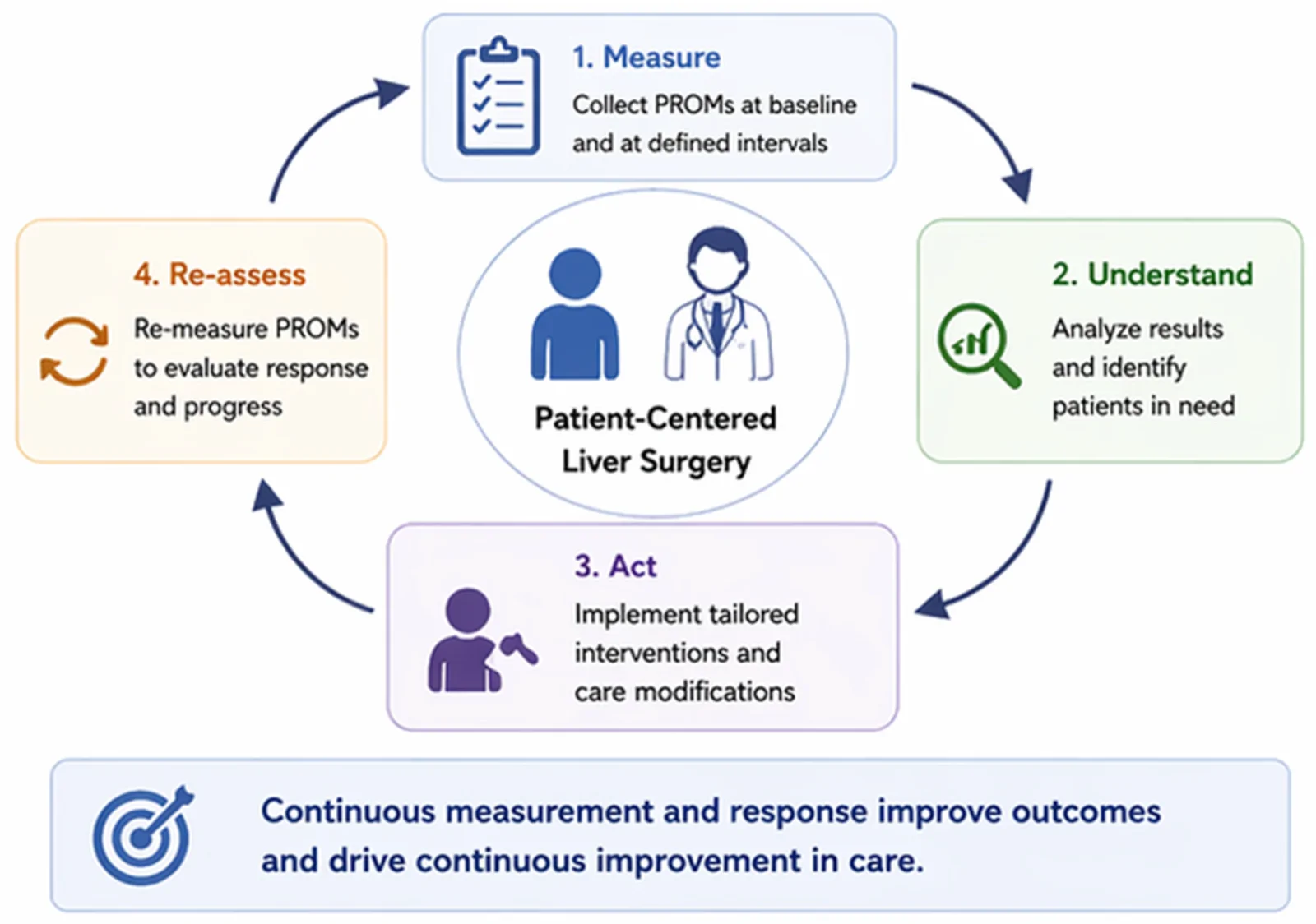
From PROMs to action: Proposed PROM-based perioperative pathway in liver surgery integrating longitudinal assessment, interpretation, intervention, and reassessment.

In this selected patient cohort, patient-reported QoL and psychological well-being scores were better at the three-month postoperative assessment compared with baseline. Given the observational design, absence of a comparison group, and heterogeneity of the study population, these changes cannot be attributed exclusively to liver resection. PROMs provide clinically meaningful information beyond conventional surgical metrics and may help identify vulnerable patients requiring tailored perioperative support. The integration of PROMs into liver surgery pathways may contribute to the development of a more patient-centered model of hepatobiliary care (Figure 3). The identification of patients at higher risk for impaired recovery may facilitate tailored postoperative monitoring and supportive interventions.

## Figures and Tables

**Table 1:** Baseline, disease, surgical characteristics and post-operative outcomes of patients.

	Variable	Value
Demographics	Age (years), median (range)	72 (39–87)
	Female, *n* (%)	29 (41)
	BMI, median (range	26.7 (18.4–42.9)
	ASA ≥ 3, *n* (%)	17 (24)
	ECOG ≥ 2, *n* (%)	3 (4)
	CCi; median (range)	7 (0–11)
Liver Status	Cirrhosis, *n* (%)	22 (30)
	Steatosis, *n* (%)	18 (25)
	Total bilirubin (mg/dL); median (range)	0.56 (0.17–6.85)
	Platelets (×10^3^/mmc): median (range)	208 (54–473)
	INR; median (range)	1.02 (0.88–1.33)
Disease	CRLM, *n* (%)	28 (39)
	HCC, *n* (%)	21 (29)
	Cholangiocarcinoma, *n* (%)	9 (12)
	Other etiologies, *n* (%)	13 (18)
	Number of lesions; median (range)	1 (1–20)
	Maximum lesion diameter (cm); median (range)	3.7 (0.9–12.5)
Surgery	Major resection, *n* (%)	19 (27)
	Minimally invasive, *n* (%)	12 (17)
	Operative time (min), median (range)	288 (90–485)
	Blood loss > 500 mL, *n* (%)	13 (18)
Postoperative outcomes	Complications at Clavien–Dindo ≥ 3, *n* (%)	4 (6%)
	Bile leak ≥ grade B, *n* (%)	5 (7%)
	PHLF, *n* (%)	0 (0)
	Mortality at 90 days, *n* (%)	0 (0)
	Length of stay (days), median (range)	8 (4–32)

**Table 2:** PROM scores at baseline and at the three-month postoperative assessment.

	Pre-Operative	Post-Operative (3 Months)	*p*-Value
Aggregate QLQ-C30, median (range)	52 (40–66)	46 (41–75)	<0.001
Aggregate QLQ-LMC21, median (range)	31 (20–44)	26 (19–43)	<0.001
Psychological aggregate QLQ-C30, median (range)	16 (8–22)	11 (8–20)	<0.001
Psychological aggregate QLQ-LMC21, median (range)	13 (6–20)	10 (6–14)	<0.001

**Table 3:** Multivariable Predictors of Postoperative QLQ-C30 Aggregate Score.

Variable	β (C30)	95% CI	*p*-Value
ASA (I–II vs. III)	2.18	−2.51–6.87	0.351
Steatosis	1.00	−4.79–6.79	0.727
Cirrhosis	−0.28	−4.95–4.39	0.904
Minor vs. major liver resection	1.53	−3.40–6.46	0.532
Minimally invasive liver surgery	2.95	−1.95–7.85	0.230
Intraoperative transfusions	3.51	−1.09–8.11	0.130
Postoperative complications at Clavien–Dindo ≥ 3	0.03	−7.47–7.52	0.994
Postoperative ascites	0.98	−8.04–9.99	0.827
Postoperative bile leak (grade B)	12.28	0.75–23.81	0.038

Note: Positive β indicates worse QoL (higher symptom burden).

**Table 4:** Multivariable Predictors of Postoperative QLQ-LMC21 Aggregate Score.

Variable	β (LMC21)	95% CI	*p*-Value
ASA (I–II vs. III)	−1.80	−4.60–1.01	0.201
Steatosis	1.36	−2.10–4.83	0.428
Cirrhosis	−0.29	−3.09–2.50	0.831
Minor vs. major liver resection	0.40	−2.64–3.43	0.792
Minimally invasive liver surgery	2.09	−0.84–5.02	0.157
Intraoperative transfusions	2.19	−0.57–4.95	0.116
Postoperative complications at Clavien–Dindo ≥ 3	1.12	−3.36–5.60	0.615
Postoperative ascites	2.00	−4.31–8.31	0.523
Postoperative bile leak (grade B)	6.24	−0.68–13.16	0.076

Note: Positive β indicates worse QoL (higher symptom burden).

## Data Availability

Data will be provided upon reasonable requests.

## Supplementary Materials

The following supporting information can be downloaded at https://www.mdpi.com/article/10.3390/cancers18172767/s1. Table S1: Postoperative PROM Comparison (QLQ-C30); Table S2: Postoperative PROM Comparison (QLQ-LMC21); Table S3: Postoperative PROM Comparison (QLQ-C30), subgroup analysis focused on psychological outcomes; Table S4: Postoperative PROM Comparison (QLQ-LMC21), subgroup analysis focused on psychological outcomes.

## Abbreviations

The following abbreviations are used in this manuscript:

QoL	Quality of Life
PROMs	Patient-Reported Outcome Measures
PREMs	Patient-Reported Experience Measures
PHLF	Post-Hepatectomy Liver Failure
FLR	Future Liver Remnant
IOUS	Intraoperative Ultrasound
PVE	Portal Vein Embolization
LVD	Liver Venous Deprivation
ALPPS	Associating Liver Partition and Portal Vein Ligation for Staged Hepatectomy
ERAS	Enhanced Recovery After Surgery
WHO	World Health Organization
HCC	Hepatocellular Carcinoma
iCCA	Intrahepatic Cholangiocarcinoma
pCCA	Perihilar Cholangiocarcinoma
CRLM	Colorectal Liver Metastases
AFP	Alpha-Fetoprotein
CEA	Carcinoembryonic Antigen
CA19-9	Carbohydrate Antigen 19-9
CT	Contrast-Enhanced Computed Tomography
MRI	Magnetic Resonance Imaging
FDG-PET	Fluorodeoxyglucose Positron Emission Tomography
ECOG	Eastern Cooperative Oncology Group
CCi	Charlson Comorbidity Index
ASA	American Society of Anesthesiologists
RFA	Radiofrequency ablation
MWA	Microwave Ablation
