# Supplementary material for: Patient-Reported Outcome Measures After Liver Resection: A Prospective Single-Center Analysis of Patient-Reported Outcomes and Predictors of Recovery
Source: Cancers (Basel). 2026 Aug 26;18(17):2767. doi: 10.3390/cancers18172767 (PMC13565504; doi:10.3390/cancers18172767)
Supplement: Supplementary file 1 [file cancers-18-02767-s001.zip › cancers-4407411-supplementary.pdf]

Supplementary Table S1. Postoperative PROMs Comparison (QLQ-C30)

	C30 POST-OP	p-value
**ASA (I-II vs III)**	**46 (43–48) vs 50 (44–53)**	**0.044**
Steatosis	45.5 (43–52) vs 48 (43–51)	0.63
Cirrhosis	47 (43–52) vs 46 (43–50)	0.83
Minor vs major resection	45 (43–52) vs 47.5 (46.5–52)	0.13
Minimally invasive surgery	47 (43–51) vs 45 (43–52.5)	0.80
**Intraoperative transfusions**	**45 (43–48) vs 52 (47–53)**	**0.011**
Postoperative complications Clavien–Dindo ≥ 3	46 (43–52) vs 45 (42.5–61)	0.85
Postoperative ascites	46 (43–50) vs 48.5 (43.5–53.5)	0.51
Postoperative bile leak (grade B)	46 (43–52) vs 45 (42.5–61)	0.83

Supplementary Table S2: Postoperative PROMs Comparison (QLQ-LMC21)

Variable	LMC21 POST-OP	p-value
ASA (I-II vs III)	27 (24–30) vs 24.5 (23–29)	0.18
Steatosis	26 (23–29) vs 27.5 (23–31)	0.34
Cirrhosis	27 (23.5–29) vs 25 (23–29)	0.43
Minor vs major resection	26 (23–29) vs 27 (24–31)	0.35
Minimally invasive surgery	25 (23–28) vs 28 (26–29)	0.09
Intraoperative transfusions	25 (23–29) vs 28 (25–29)	0.10
Postoperative complications Clavien–Dindo ≥ 3	26 (23–29) vs 27 (25–35)	0.63
Postoperative ascites	27 (23–29) vs 24 (22–29)	0.56
Postoperative bile leak (grade B)	26 (23–29) vs 27 (24.5–35)	0.68

Supplementary Table S3. Postoperative PROMs Comparison (QLQ-C30), subgroup analysis focused on psychological outcomes.

	C30 POST-OP	p-value
ASA (I-II vs III)	11 (9–12) vs 11 (9–13)	0.62
Steatosis	10.5 (9–12) vs 11.5 (9–13)	0.58
Cirrhosis	11 (9–13) vs 11 (9–12)	0.45
**Minor vs major resection**	**10 (8–12) vs 12 (11–13)**	**0.008**
Minimally invasive surgery	11 (9–12) vs 9.5 (9–13)	0.73
**Intraoperative transfusions**	**10 (8–12) vs 12 (9–13)**	**0.039**
Postoperative complications Clavien–Dindo ≥ 3	11 (9–12) vs 8.5 (8–13.5)	0.35
Postoperative ascites	10 (9–12) vs 11.5 (10–12.5)	0.49
Postoperative bile leak (grade B)	11 (9–12) vs 9 (8.5–13.5)	0.51

Supplementary Table S4: Postoperative PROMs Comparison (QLQ-LMC21), subgroup analysis focused on psychological outcomes.

Variable	LMC21 POST-OP	p-value
**ASA (I-II vs III)**	**11 (9–12) vs 9 (8–11)**	**0.043**
Steatosis	9.5 (9–11) vs 10.5 (8–14)	0.37
Cirrhosis	10 (9–12) vs 9 (8–11)	0.60
**Minor vs major resection**	**9 (7–11) vs 11 (9–12.5)**	**0.035**
Minimally invasive surgery	9 (8–12) vs 10.5 (8.5–11)	0.78
Intraoperative transfusions	9 (8–11) vs 11 (9–11)	0.15
Postoperative complications Clavien–Dindo ≥ 3	10 (8–11) vs 9.5 (9–12)	0.67
Postoperative ascites	10 (8–11) vs 9.5 (8.5–10.5)	0.75
Postoperative bile leak (grade B)	10 (8–11) vs 9.5 (8.5–12)	0.84
